# Fractures of the acetabulum: from yesterday to tomorrow

**DOI:** 10.1007/s00264-020-04806-4

**Published:** 2020-09-22

**Authors:** Matej Cimerman, Anže Kristan, Marko Jug, Matevž Tomaževič

**Affiliations:** Traumatology Department, Division of Surgery, University Medical Centre Ljubljana, Ljubljana, Slovenia

**Keywords:** Acetabulum, Fracture, Planning, Approaches, History

## Abstract

**Purpose:**

The aim of this article is to present history, state of the art, and future trends in the treatment of acetabular fractures.

**Methods:**

Review of recent and historical literature.

**Results:**

Acetabular fractures are difficult to treat. The first descriptions of this injury already appeared in ancient Greek history, but intensive development started in the second half of the twentieth century after Judet and Letournel’s seminal work. Their classification is still the gold standard today. It is actually a pre-operative planning system and is used to determine the most appropriate surgical approach. The therapy of choice for dislocated fractures is open reduction and internal fixation. Recent modern techniques based on high-tech computerized planning systems and 3D printing have been successfully integrated into orthopaedic trauma practice.

**Conclusion:**

There is no ideal surgical approach for acetabulum fracture treatment, so new approaches have been developed in recent decades. The best outcome series have shown good or excellent results, between 70 and 80%.

## Introduction

Acetabular fractures are among the most demanding injuries treated by orthopaedic trauma surgeons. The incidence is three patients/100,000/year [1], so even in a busy European trauma centre, the case load is relatively small and it is difficult to gain enough experience. There are a lot of challenges in acetabular fracture surgery. The 3D morphology of the fracture is complex, and the choice of surgical approach is not always straightforward. Surgical approaches are demanding, and anatomical reduction, which is the most decisive factor for good long-term outcome [2], can pose difficulties, even for the most experienced surgeons. In this review article, we summarize the development and state of the art of the surgical treatment of acetabular fractures.

## Historical overview

The first and for a long time the only description of acetabular fracture came from Homer’s Iliad, written during the eighth century BC. The poetic description of acetabular injury in Iliad is as beautiful as it is accurate and should be read by every acetabular surgeon: “Just as Diomedes hefted a boulder in his hands, a tremendous feat — no two men could hoist it, weak as men are now, but all on his own he raised it high with ease, flung it and struck Aeneas’s thigh where the hip bone turns inside the pelvis, the joint they call the cup — it smashed the socket, snapped both tendons too, and the jagged rock tore back the skin in shreds. The great fighter sank to his knees, bracing himself with one strong forearm planted against the earth, and the world went black as night before his eyes” [3].

Besides the poetic beauty, there are some astonishing facts: exactly the same mechanism of injury; i.e. a direct blow to the greater trochanter was described in an experimental study by Pearson 2800 years later [4], and the description of the pain is astonishingly real.

Four centuries after Homer, Hippocrates described injuries around the acetabulum under the common term “hip dislocations” [5] because it was impossible to differentiate between an acetabulum fracture and hip dislocation by clinical examination alone. Acetabular fractures were considered to be hip dislocations until the nineteenth century. The first detailed description of an acetabular fracture before the discovery of X-rays (W. Roentgen 1896) was made by Sir Astley Cooper in 1818. The diagnosis was made on autopsy. Cooper described a fracture of the innominate bone with central dislocation [6]. Schroeder, in a systematic article, analyzed 49 cases of fractures with central dislocations [6]. He stated that these are severe injuries caused by high energy, with a mortality rate of around 30%. In the first half of the twentieth century, the treatment of choice was conservative therapy. Urist reported results after treatment of fracture dislocations of the hip in military personnel [7]. He suggested open reduction and internal fixation for dislocated fractures. Eliot and Knight reported on operatively treated central acetabular fractures [8, 9]. Despite these reports, the majority of patients were treated conservatively. Rowe and Lowell published an end-result study on 99 patients and recommended conservative therapy [10]. In 1964, Eichenholtz described the current situation: “There are wide differences in opinion on the relative merits of the two forms of therapy, and among the advocates of open treatment there is no agreement as to the type of surgical procedure indicated” [11]. In such an atmosphere, Judet and Letournel began their revolutionary work on this topic. The biggest stimulation to their study was their “extreme disappointment” [12] with the results of conservative treatment. In 1963, they published their classical article with the informative title “Fractures of the Acetabulum, Classification and Surgical Approaches for Open Reduction” [13]. They described their classification, which enables understanding of the complex 3D geometry of a fracture, allowing a logical choice of operative approach. They also developed two new surgical approaches. They recommended open reduction and internal fixation for all displaced acetabular fractures [14]. It took several more years for this idea to be widely accepted. There was a lot of scepticism about the operative treatment of acetabular fractures in North America in the 1970s, and some literature still advocated conservative treatment [15, 16]. However, Judet and Letournel continued the dissemination of their ideas. They published their textbook in English translation in 1981 and a revised edition in 1993 [12, 17]. These textbooks became very popular and are still today considered to be “the bible” of acetabular surgeons. Letournel was active as an educator. He was a guest of honour at AO (Arbeitsgemeinschaft für Osteosynthesefragen) courses in Davos in 1982, and started courses of pelvic surgery in Paris and North America. In the 1980s, he trained a group of five North American surgeons, who were later called the acetabular club [12]. They spread Letournel and Judet’s teachings in North America and also worldwide. One of them, Joel Matta, later published the largest single surgeon outcome study of operatively treated acetabular fractures [18]. The era of modern acetabular surgery had started. At our institution, we performed the first documented internal fixation of a posterior wall fracture in 1965, followed by systematic development of pelvic and acetabular surgery.

## Classification, decision-making, and planning

Judet and Letournel introduced their classification of acetabular fractures in 1964 and made a slight refinement in 1974 [14, 17]. The classification is the gold standard, has definitely stood the test of time, and is the preferred classification for the majority of orthopaedic trauma surgeons [19]. Before their classification, the morphology of acetabular fractures was poorly understood. Letournel wrote that the choice of operative approach was more or less a “toss-up” [12]. The choice of approach is important, because no single surgical exposure allows convenient access to both columns without consequences. He realized that the problem was not the operative approach per se, but the inability “to grasp the precise outline of the fracture from traditional AP radiographs” [12]. After an intensive study of the anatomy of the innominate bone, they developed the concept of two columns, which is the cornerstone of their classification. The beauty of their classification is that it is in fact a pre-operative planning system and is used to determine the most appropriate treatment, especially the right surgical approach. From three standard X-ray projections (AP, iliac, and obturator) and four lines (iliopectineal, ilioischial, both walls), it is possible to understand the 3D morphology of fractures and classify them into five elementary and five associated types. Elementary fractures are those in which a part or all of one column is detached (posterior column, posterior wall, anterior column, anterior wall, transverse), and associated fractures include at least two of the elementary forms (T-shaped, transverse with posterior wall, posterior column with posterior wall, anterior column with posterior hemitransverse, and both columns) [12].

Intra- and interobserver reliability was high in an expert group but lower with less-trained surgeons [20–22], giving the impression that the classification system is too difficult and complex. The difficulty and complexity probably came from the challenging nature of acetabular fractures, rather than being inherent to the classification system [19]. The learning curve can be long, but it is possible to shorten it using an algorithmic approach [23, 24], 3D CT, and modern 3D modules [25–27].

**Fig. 1:**
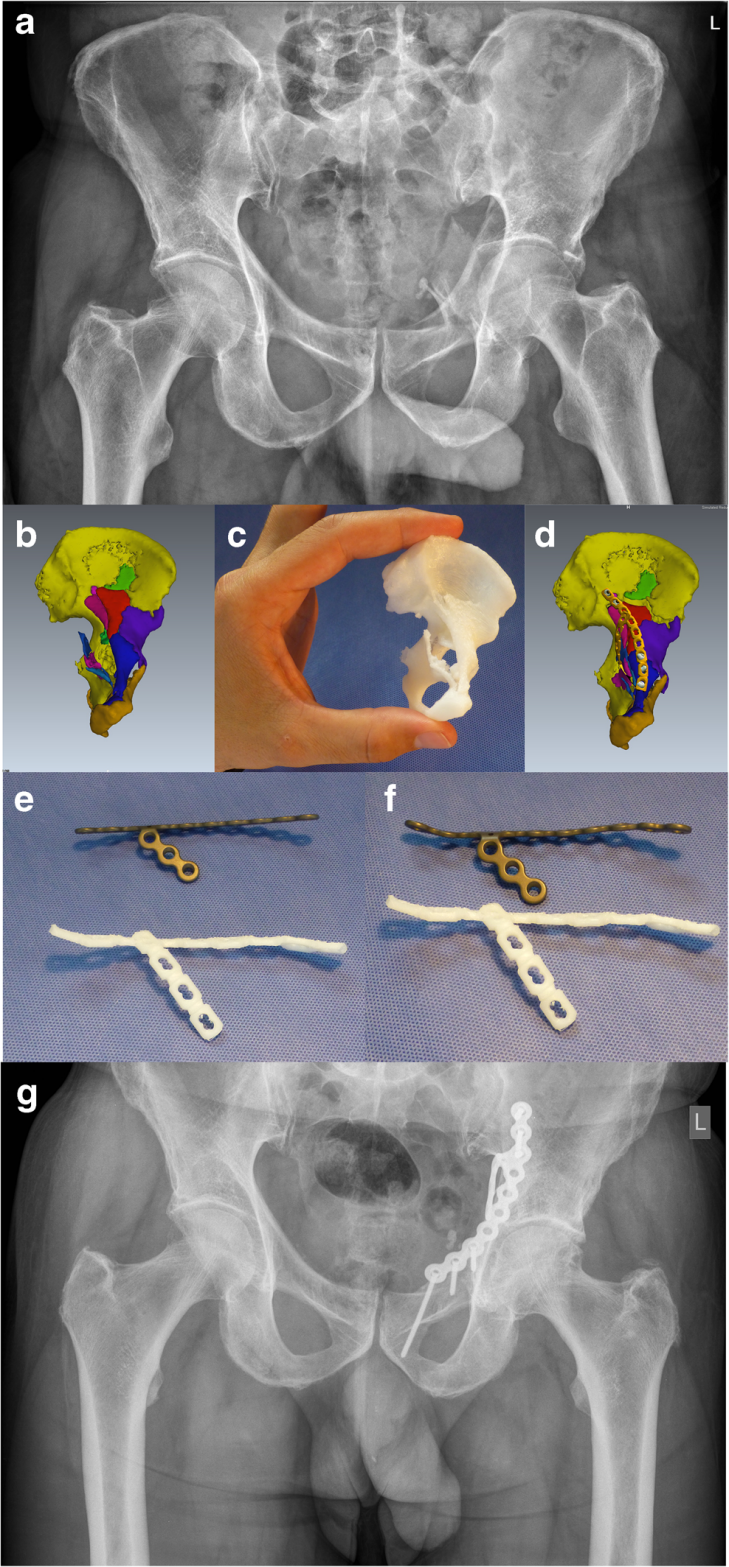
Process of 3D planning: **a** comminuted anterior column fracture of the acetabulum in a 65-year-old male, **b** fracture segmentation using pre-operative planning software, **c** a reduced size 3D-printed model enables visual and tactile understanding, **d** virtual reduction and individual designed shape of the plate, **e**, **f** individual designed 3D-printed plastic plates according to virtually reduced fracture serves as a template to contour reconstruction plates, **g** result 3 years after open reduction and internal fixation (the Pararectus surgical approach was used)

Accurate classification of acetabular fractures is therefore possible on the basis of conventional X-rays and is the first step in decision-making. The second step is a 2D CT scan, which can detect many important details that are not included in the classification: small intra-articular fragments, impactions, quantifying intra-articular step and gap, discrete fractures of the femoral head, and subluxation [28, 29]. The whole imaging process is concluded with 3D CT. The 3D is easily correlated with a plain radiograph and provides a unique perspective of the fracture [30]. A decision on treatment is now possible. The indications for operative treatment according to Letournel are straightforward: all dislocated acetabular fractures [12]. Nowadays, we have some evidence for quantifying a dislocation. Jenssen et al. demonstrated in a study of 186 hips that conservatively treated acetabular fractures with less than 2-mm displacement had 94% survival after 10 years [31]. Matta found 91% good or excellent results in operated patients with less than 3-mm displacement [32]. Other generally accepted indications for operative treatment include as follows: the acetabular articular surface is intact in the superior 10 mm of the joint on CT evaluation, congruent hip joint [33], intra-articular fragments, and unstable hip. The ideal timing for operation according to Letournel is from two to six days after the injury [12]. Mears confirmed this interval, demonstrating that anatomic reduction is significantly decreased after a delay of more than 11 days [34]. A surgeon therefore has only a short time for pre-operative planning. Jeffrey Mast, a legend of preoperative planning in fracture surgery and the co-author of a best-selling textbook about planning [35] said: “The construction of ships, automobiles… involves sophisticated plans rendered in drawings to the most minute detail. These systems are designed to discover flaws in the project beforehand. A similar system in orthopaedic surgery is also possible” [36]. Judet and Letournel had done exactly the same 20 years earlier and one can admire the precise drawings of complex acetabular fractures in their textbook [12]. Planning enables an understanding of the fracture lines in detail, the choice of the right operative approach, and an outline of surgical tactics. Modern techniques based on high-tech computerized planning systems have been successfully integrated into orthopaedic trauma practice [37–40]. In 2007, we introduced an experimental computer program at our institution, for the virtual operation of acetabular fractures based on real data [37]. The module consists of a 3D viewing tool and a simulation tool based on DICOM data from 1.5-mm CT slices. Segmentation of each fracture fragment can be performed in a different colour. Manipulation of the fragments in 3D, with virtual reduction and fixation, is possible. The plates are automatically contoured to reduce the acetabulum. The direction and length of the screws can be controlled. Intra-operative fluoroscopy can also be simulated. It is possible at the end to compare the planned and real procedures. Surgeons are generally satisfied with virtual 3D planning [37, 41]. Chen et al. demonstrated a better clinical outcome in patients operated with planning and 3D modelling than in the conventional group [42] and Citak described better reduction on plastic models after 3D virtual planning [38]. The next logical step is to connect 3D virtual planning with 3D printing, which means creating a real 3D model from a digital image [43]. The digital image can be converted to a.stl (StereoLitography) file and sent to a 3D printer. This technology is becoming more and more accessible and affordable and is already a mainstream in many fields of medicine [43]. A 3D-printed model of a fractured acetabulum allows the surgeon tactile and visual understanding of the specific fracture. Models also allow pre-operative simulations of difficult fracture reduction and fixation and greatly facilitate education [44]. The plates can be shaped according to a bone model pre-operatively, better to match the reduced fracture during the surgery. [45]. In our institution, we plan the fixation on a virtual model and bend the plates according to the reduced acetabulum. We then print models of the plates from plastic and use them as templates for contouring the real plates. We have found this technique to be very useful (Fig. 1). Technically it is now possible to print patient-specific custom-made plates from titanium. We have also demonstrated that different surgeons designed different implants for the same fracture, so these implants were patient and surgeon specific [46]. The next step can therefore be the printing of real implants according to the patient-specific anatomy, surgeon’s aspirations, and optimal biomechanical properties.

## Surgical approaches

The functional outcome of operatively treated acetabular fractures depends directly on the accuracy of reduction [12, 18, 32], and the most decisive factor for performing the best reduction possible is the right choice of surgical approach [47]. The approach in acetabular fracture surgery poses specific problems: first of all, the acetabulum lies deep and is covered by important neurovascular structures, which makes the approach technically demanding and sometimes risky. Secondly, no single approach allows access to the entire acetabulum [48]. Judet and Letournel were aware of this more than half a century ago, when they started to understand the complex geometry of acetabular fractures. For posterior fractures, they used the Kocher-Langenbeck approach and they looked for an approach for the anterior column. After a serious study in an anatomy lab, Letournel introduced the ilioinguinal approach (IL) and started to use it in 1965 [12]. The approach is composed of three windows. The first window provides access to the internal iliac fossa and sacroiliac joint. The second or middle window grants access to the pelvic brim and quadrilateral surface from above, and the third window, medial to the iliac external vessels, gives access to the superior pubic ramus. The approach allows a complete access to the anterior column [12]. IL is extensive and technically demanding and needs a long learning curve. When the authors began also to treat delayed cases, they felt the need for simultaneous exposure of both columns, and ten years later, they introduced the extended iliofemoral approach. This approach enables access to the whole external surface of the iliac bone and is anterior limited by the iliopectineal eminence [12]. The approach is very extensile and demanding. These three approaches became the gold standard for acetabular fracture surgeons and have remained so until today. Despite favourable long-term results for experts using the mentioned approaches [12, 18], the development of new approaches and improvement of classic approaches have been obvious in recent decades. First of all, there has been a decline in using extensile approaches. While in his original series, Letournel used an extended iliofemoral approach in 14% of patients [49] and Matta in 22% [50], and in a meta-analysis from 1966 until 2004, extensile approaches were still used in 17% of cases [51], more recent studies have shown a marked decline in the use of extensile approaches to 4% [52] and to 0.4% in a study from UK [53]. Extensile approaches are associated with a prolonged operative time, blood loss, high percentage of heterotopic ossifications, and wound complications, even in the most expert hands [54–56]. New generations of acetabular surgeons use extensile approaches less and less. If a surgeon is not comfortable with large approaches, he is not likely to use them even in rare cases. This is a self-accelerating phenomenon and it is possible that young acetabular surgeons in future will see extensile approaches only in textbooks and cadaveric labs [47]. It is perhaps easier to be familiar with anterior and posterior approaches and combine them in complex cases. Equally, improvements in the posterior approach, the development of new anterior approaches, and better pre-operative planning have pushed the limits of a single approach forward.

Moed described a modified Gibson approach to the posterior column [57]. The approach is similar to Kocher-Langenbeck and differs only in its proximal dissection: the interval between the tensor fasciae latae and gluteus maximus is developed and the gluteus maximus is displaced from its anterior border without splitting. This protects the neurovascular supply to the anterior part of the muscle. The approach also enables better visualization of the anterosuperior part of the acetabulum and can be combined with trochanter flip osteotomy. Gautier et al. from the Bernese group studied the anatomy of the medial circumflex artery in detail [58]. They demonstrated a constant course of the deep branch of the medial femoral circumflex artery in the extracapsular segment. This pivotal work enabled a safer approach to the posterior column and also makes possible trochanter flip osteotomy, with or without surgical dislocation of the hip [59]. Ganz published a unique series of 213 surgical dislocations of the hip without a single avascular necrosis of the femoral head [59]. Trochanteric flip osteotomy without surgical dislocation of the hip allows safe exposure of the posterosuperior and superior parts of the posterior column without damage to the abductor muscles [60]. The osteotomy is balanced by the opposite pull of the gluteus medius and vastus lateralis muscle. Surgical dislocation of the hip makes possible direct visual control of the acetabulum during reduction and fixation [61, 62] and can be used in the surgical treatment of femoral head fractures [63].

In 1993 and 1994, Hirvensalo et al. [64] and Cole and Bolhofner [65] independently describe a new anterior approach, which is now called the anterior intra-pelvic approach (AIP). In AIP, the recti muscles are split at the midline and further dissection is performed extraperitoneally direct to the posterior aspect of the pubis to the quadrilateral surface. The iliopectineal fascia is released from the pelvic brim, the femoral vessels are moved anterior, and the inner surface of the true pelvis is exposed. The main difference of IL is that in AIP, there is no medial window and the surgeon stands on the opposite side of the fracture and “looks in” while in IL, the surgeon remains on the injured side and “looks over” [47]. If the fracture extends to the iliac wing, it is possible with AIP to open the first (iliac) window of the ilioinguinal approach and use it for reduction and fixation of the iliac wing [65]. The new AIP approach has become more and more popular worldwide. There are several reasons for this rising popularity: it is potentially less invasive than IL and enables excellent visualization of the entire pelvic brim from the pubic body to the sacroiliac joint, including direct visualization of the quadrilateral plate [66]. The new approach is therefore very suitable for anterior fractures, including central luxation, which is essentially a typical geriatric fracture pattern. These fracture patterns are more and more frequent because of the rapidly growing elderly population [67], and the treatment strategy should be adapted to this. Dissemination of the AIP approach has also encouraged the development of new instruments and implants [68]. It is difficult to compare surgical approaches, but a study by Rocca et al. and meta-analysis by Meena et al. both favour AIP over IL [69, 70]. The Bernese group recently contributed a new anterior approach called Pararectus [71, 72]. The approach combines the advantages of intra-pelvic and ilioinguinal approaches. It is technically demanding and, although the first results are promising [73], further studies are needed.

It would be ideal for an acetabular surgeon to master all the approaches (classic and novel), because they are complementary not competitive and enable specific details for specific fractures. However, in practice, because of the relatively low case load, the majority of surgeons master a limited number of approaches. The approach should therefore be chosen according to fracture pattern, soft tissue status, and the individual preference and skill of the surgeon. From 2002 to 2020, we operated 361 acetabular fractures; an intra-pelvic approach was used in 142 (39%), Kl or Gibson with or without trochanteric flip in 184 (51%), a combination of anterior and posterior in 23 (6%), Pararectus in four (1%), and ilioinguinal in three (1%) (unpublished data).

## Outcome

Numerous outcome studies have shown good or excellent results, between 70 and 80%. The best results and the biggest series were presented by Letournel [12] and Matta [18], 491 and 816 hips, respectively. Matta also analyzed the survival of hips, which was 85% for ten years and 79% for 20 years. These long-term results appear largely unchanged and still represent the gold standard. It is a question why the results from Letournel have not really been bettered, despite the intensive development of acetabular surgery. One reason is a significant change in age and fracture pattern. The number of elderly patients and anterior fracture patterns has risen significantly in recent decades [52, 67]. Anterior patterns, especially anterior wall fractures, have the worst long-term results [18, 51] and to fix osteoporotic bone is also harder. It is necessary to wait some more years for long-term results by surgeons from the new generation. However, in the last decade, there has been a significant drop in iatrogenic nerve injuries and surgery is performed earlier [74].

## Conclusions

The modern era of acetabular surgery started in the 1960s after Judet and Letournel’s classic work. There has been intensive development in recent decades. New imaging modalities, including 3D CT, have helped in understanding complex fracture patterns. Computer technology has enabled precise pre-operative planning. The most recent technology has made real even a tactile and visual feeling of the fracture via printed models. Surgeon and patient-specific implants will soon be able to be printed. The classic posterior approach has been refined, and new anatomic knowledge allows safe surgical dislocation of the hip. Modern anterior approaches are promising and are very useful, especially in rapidly growing geriatric anterior fractures. Even in the best hands, good results do not exceed 80%, leaving a lot of room for improvement.

## Data Availability

This is a review paper. Data stated by our hospital is available by the request to the authors.
